# MRI-Defined Osteonecrosis Extent After Locked Plate Fixation of Proximal Humeral Fractures: Correlation with Shoulder Motion and Radiographic Severity

**DOI:** 10.3390/jcm15166491

**Published:** 2026-08-21

**Authors:** Ahmet Serhat Aydin, Dağhan Koyuncu, Alper Şükrü Kendirici, Mert Ballı, Ali Erşen, Görkem Durak, Memduh Dursun

**Affiliations:** 1 Department of Orthopedics and Traumatology, Faculty of Medicine, Akdeniz University, 07070 Antalya, Türkiye; 2 Department of Orthopedics and Traumatology, Istanbul Physical Therapy and Rehabilitation Research and Training Hospital, 34098 Istanbul, Türkiye; daghanorto@gmail.com; 3 Department of Orthopedics and Traumatology, Faculty of Medicine, Istanbul University, 34093 Istanbul, Türkiye; 4 Department of Radiology, Faculty of Medicine, Istanbul University, 34093 Istanbul, Türkiye

**Keywords:** proximal humeral fracture, osteonecrosis, avascular necrosis, magnetic resonance imaging, shoulder motion, locked plate fixation, Cruess classification, functional outcome

## Abstract

**Background/Objectives:** Humeral head osteonecrosis is a recognised complication of locked plate fixation for proximal humeral fractures, yet the significance of necrotic lesion extent is poorly characterised. This study quantified MRI-defined osteonecrosis extent and its associations with radiographic severity, shoulder motion, and functional outcomes. **Methods**: Twenty-five patients with Neer three- or four-part proximal humeral fractures treated by locked plate fixation between 2010 and 2022 were retrospectively reviewed. Shoulder range of motion, Constant score, American Shoulder and Elbow Surgeons (ASES) score, and visual analogue scale (VAS) pain were assessed. Osteonecrosis severity was graded by the Cruess classification. Necrotic extent was quantified on MRI using an angle-based method on coronal and sagittal images; associations were assessed by simple linear regression among patients with confirmed osteonecrosis (*n* = 12). **Results:** At a mean follow-up of 85.4 ± 34.1 months, osteonecrosis was present in 12 of 25 patients (48%). Affected patients had lower shoulder abduction (96.7 ± 27.1° vs. 121.8 ± 29.2°; mean difference −25.1°, 95% CI −47.9 to −2.3; *p* = 0.031), internal rotation (48.3 ± 15.9° vs. 66.4 ± 15.5°; mean difference −18.1°, 95% CI −32.1 to −4.1; *p* = 0.014), and external rotation (36.7 ± 16.7° vs. 51.8 ± 26.4°; mean difference −15.1°, 95% CI −29.5 to −0.7; *p* = 0.041). Functional scores did not differ. Among patients with osteonecrosis (*n* = 12), greater necrotic angle correlated with higher Cruess stage and reduced abduction (β = −0.141; 95% CI −0.259 to −0.024; *p* = 0.020). **Conclusions**: MRI-defined osteonecrosis extent correlates with radiographic severity and reduced shoulder motion after locked plate fixation. Larger necrotic lesions are linked to decreased abduction, with no clear relationship to global functional scores. Quantitative MRI may provide useful information on the structural severity of post-traumatic humeral head osteonecrosis.

## 1. Introduction

Among adults, proximal humeral fractures represent approximately 4–5% of all skeletal injuries and rank among the most frequently encountered upper-extremity fractures [1,2]. Within this spectrum, Neer three- and four-part configurations present a particularly demanding surgical problem owing to significant displacement, metaphyseal fragmentation, and vulnerability of the humeral head blood supply [3]. Operative stabilisation is often necessary, and locked plate fixation has established itself as a widely adopted technique for these complex injuries [4].

Despite advances in implant design and surgical technique, post-operative complications remain a meaningful clinical concern. Avascular necrosis (AVN) of the humeral head is among the most consequential, given its potential to cause articular collapse, secondary osteoarthritis, pain, and progressive loss of shoulder function [5,6]. Published rates after proximal humeral fracture fixation vary substantially across series, owing to heterogeneity in fracture severity, vascular disruption, reduction adequacy, construct stability, and duration of follow-up [5,6,7].

Research into post-traumatic humeral head osteonecrosis has identified several contributing factors. Fracture pattern and integrity of the medial calcar hinge are critical determinants of humeral head viability after intracapsular injuries [5]. Fracture classification systems have also been evaluated for their utility in estimating osteonecrosis risk [8]. Nevertheless, prior investigations have largely focused on whether AVN develops rather than on the extent of necrotic humeral head involvement and its functional consequences.

The relationship between structural AVN severity and shoulder function is not well defined. Inferior outcomes following post-traumatic humeral head osteonecrosis have been documented in some cohorts [9], whereas other investigators have shown that satisfactory function can coexist with radiographic necrosis [10]. This dissociation implies that tissue destruction and functional impairment do not necessarily progress in parallel.

MRI affords detailed evaluation of bone marrow viability and osteonecrotic boundaries after fracture fixation [11]. Angle-based measurements—originally developed for femoral head osteonecrosis to quantify lesion size and collapse risk [12]—have since been applied to humeral head osteonecrosis [13]. Optimised postoperative MRI protocols have improved image quality by mitigating metal artefacts from fixation hardware [14]. Despite these advances, MRI-based quantification of AVN extent after locked plate fixation of proximal humeral fractures remains limited.

The purpose of this study was to quantify the extent of humeral head osteonecrosis using MRI after locked plate fixation of three- and four-part proximal humeral fractures and to investigate its relationship with radiographic severity, shoulder motion, and functional outcomes.

## 2. Materials and Methods

### 2.1. Study Design and Patient Selection

This ambispective cross-sectional study retrospectively identified consecutive patients who had undergone locked plate fixation for proximal humeral fractures at a tertiary referral centre between January 2010 and December 2022. Following Institutional Review Board approval (Istanbul University Faculty of Medicine IRB, Approval No: E-29624016-050.99-917586; approved 27 May 2022), eligible patients were prospectively contacted and recalled for a single final follow-up assessment including clinical examination, radiography, and MRI. Data collection was completed in December 2022. All procedures conformed to the ethical standards of the 1964 Declaration of Helsinki and its later amendments.

Eligible patients were adults (age > 18 years) with a Neer three- or four-part proximal humeral fracture [15] treated by open reduction and internal fixation with a locking plate. Fixation was performed using the PHILOS proximal humeral locking plate (DePuy Synthes, Zuchwil, Switzerland) in all cases. Medial calcar screws were used in 18 of 25 patients (72%); suture augmentation was not routinely employed. Exclusion criteria were as follows: follow-up shorter than 6 months; pathological fracture; nonunion; revision osteosynthesis; primary hemiarthroplasty or reverse shoulder arthroplasty; postoperative loss of medial calcar continuity; incomplete radiological or clinical data; and refusal to participate.

**Figure 1:**
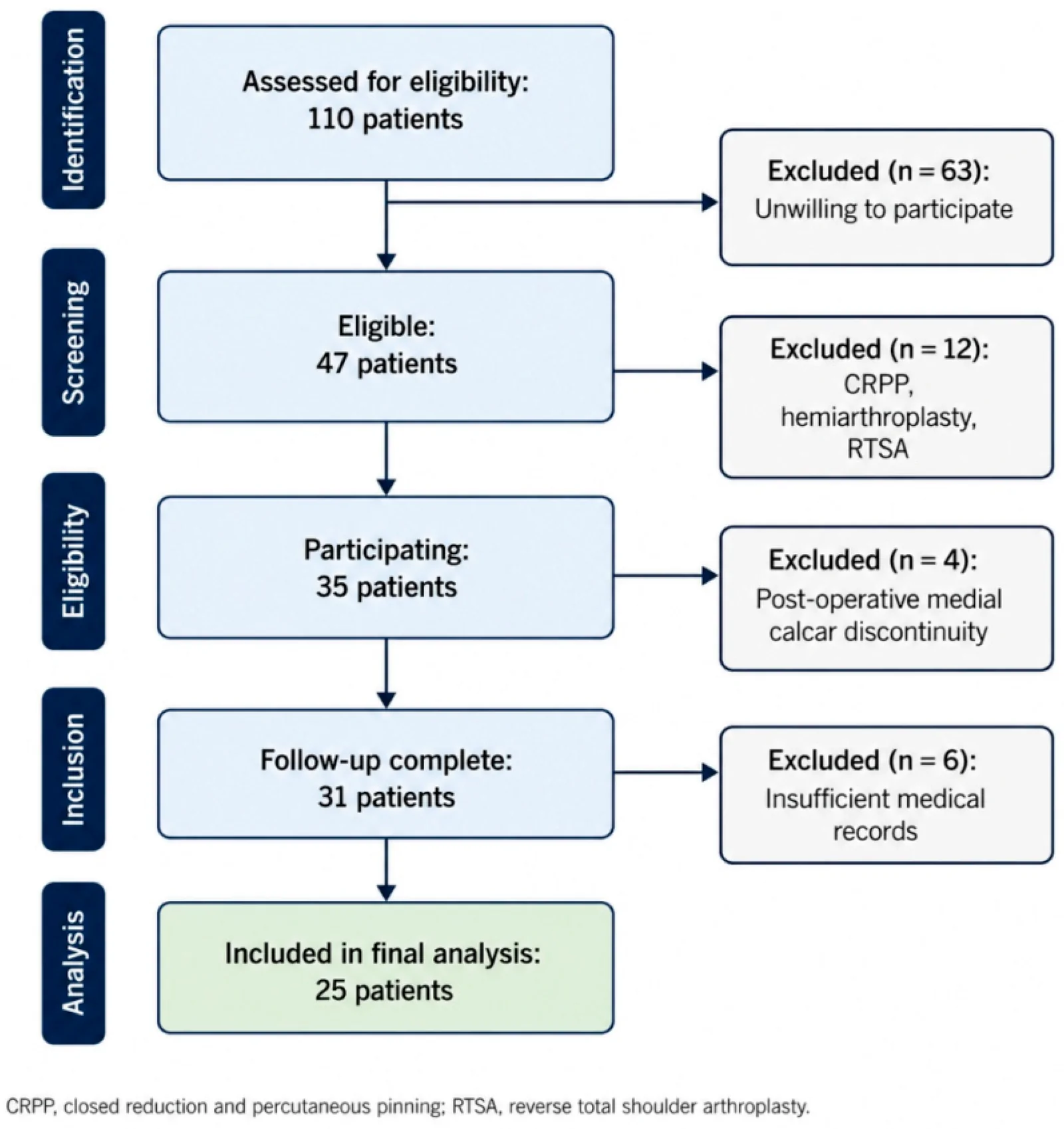
Flow diagram illustrating patient identification, application of inclusion and exclusion criteria, and final cohort selection.

Of 110 patients identified from operative records, 25 met all inclusion criteria and were included in the final analysis (Figure 1). Sixty-three patients declined participation or could not be contacted; the remaining twenty-two were excluded on clinical grounds (see Figure 1). The high non-participation rate is acknowledged as a potential source of selection bias and is discussed in Section Limitations.

### 2.2. Clinical Assessment

At the final follow-up visit, clinical examination and MRI were performed on the same day. Clinical assessment, including goniometric range-of-motion measurements and functional scoring, was completed before image acquisition. All patients underwent standardised assessments by a senior orthopaedic surgeon who recorded goniometric measurements and outcome scores prior to MRI examination, thereby maintaining blinding to imaging results at the time of clinical assessment. Active shoulder range of motion, including abduction, internal rotation at 90° abduction, and external rotation, was measured with a goniometer. Functional outcomes were assessed using the Constant score [16], the American Shoulder and Elbow Surgeons (ASES) score [17], and a visual analogue scale (VAS) for pain [18].

### 2.3. Radiological Evaluation

Standardised anteroposterior and oblique shoulder radiographs were obtained at final follow-up. Osteonecrosis severity was graded according to the Cruess classification [19]. Radiographic and MRI evaluations were independently performed by two experienced musculoskeletal radiologists (G.D. and M.D.) blinded to all clinical findings and functional outcome data. Disagreements were resolved by consensus. Inter-observer reliability for the necrotic angle measurements was assessed using the intraclass correlation coefficient (ICC, two-way mixed model, absolute agreement) calculated from independent reads on all 12 patients with confirmed osteonecrosis prior to consensus discussion. ICC for the coronal necrotic angle was 0.851 (95% CI 0.545–0.952), for the sagittal angle 0.854 (95% CI 0.560–0.954), and for the total necrotic angle 0.864 (95% CI 0.584–0.957), indicating good-to-excellent inter-observer agreement.

### 2.4. MRI Protocol and Quantification of Osteonecrosis Extent

MRI examinations were performed at final follow-up using a dedicated shoulder coil. The imaging protocol included coronal oblique T1-weighted, coronal oblique fat-suppressed T2-weighted or proton density-weighted, axial fat-suppressed proton density-weighted, and sagittal oblique sequences for assessment of bone marrow signal, subchondral integrity, and humeral head morphology. To minimise implant-related susceptibility artefacts, fast spin-echo–based sequences with thin-slice acquisition and high-bandwidth parameters were employed. A metal artefact reduction sequence (MARS) protocol was available and applied in 19 of 25 patients; in the remaining 6 patients examined on older 1.5 T equipment, standard fast spin-echo sequences were used. No examinations were excluded from quantitative analysis due to insufficient artefact reduction; however, the adequacy of necrotic boundary visualisation was confirmed independently by both radiologists before measurements were accepted.

**Figure 2:**
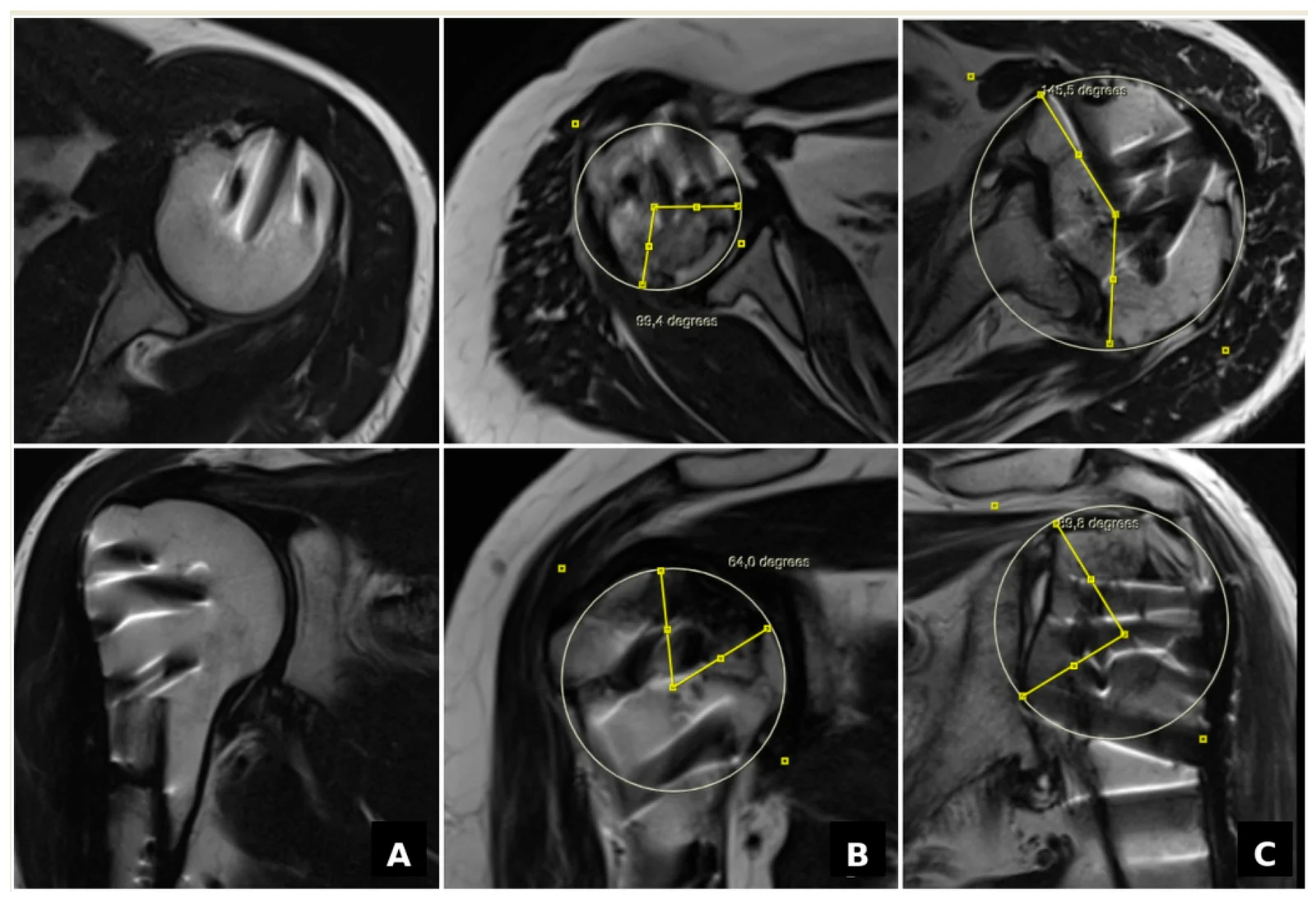
MRI-based quantification of humeral head osteonecrosis extent after locked plate fixation of proximal humeral fractures. (**A**) Fifty-eight-year-old man, Cruess stage I osteonecrosis. Mid-coronal T1-weighted image demonstrating preserved humeral head morphology without measurable necrotic angle. (**B**) Forty-seven-year-old woman, Cruess stage III osteonecrosis, total necrotic angle 163° (coronal 99°, sagittal 64°). Mid-axial fat-suppressed proton density-weighted image showing the necrotic angle measurement. (**C**) Sixty-three-year-old man, Cruess stage IV osteonecrosis, total necrotic angle 235° (coronal 146°, sagittal 90°). Representative coronal and sagittal images demonstrating a large necrotic lesion with advanced structural involvement. Total necrotic angle was calculated as the sum of the coronal and sagittal plane measurements. Yellow lines and circles indicate the best-fit circle and angle measurement lines used to quantify the necrotic angle on each imaging plane.

Osteonecrosis extent was quantified using a modified angle-based technique adapted from established assessment methods [12,13]. Mid-coronal and mid-axial images through the centre of the humeral head were selected. The humeral head centre was determined geometrically using a best-fit circle, and lines were drawn from this centre to the opposing margins of the necrotic region. The angle subtended by these lines defined the necrotic angle on each imaging plane. The total necrotic angle was calculated as the sum of the sagittal and coronal measurements (Figure 2). Patients with MRI evidence of osteonecrosis were subsequently categorised by total necrotic angle: <100°, 100–200°, and >200°.

### 2.5. Statistical Analysis

Analyses were performed using IBM SPSS Statistics (version 25.0; IBM Corp., Armonk, NY, USA). Continuous variables are reported as mean ± standard deviation with mean differences and 95% confidence intervals; categorical variables as frequencies and percentages. Distributional normality was assessed with the Shapiro–Wilk test. Between-group comparisons (osteonecrosis vs. no osteonecrosis) used the independent-samples t-test; mean differences with 95% confidence intervals are reported alongside *p*-values. Given the exploratory nature of the analysis and the small sample, no correction for multiple comparisons was applied to the primary between-group comparisons; readers should interpret the results in this context. Differences across necrotic-angle subgroups (*n* = 5, 4, 3) were examined with one-way ANOVA; these analyses are reported descriptively and should be considered hypothesis-generating only given the very small subgroup sizes. Simple linear regression was used to examine associations between total necrotic angle and clinical outcomes among patients with confirmed osteonecrosis (*n* = 12); a sensitivity model incorporating follow-up duration as a covariate was additionally fitted for shoulder abduction. Statistical significance was set at *p* < 0.05.

Applying a Bonferroni correction for the eight comparisons in Table 1 (adjusted threshold *p* < 0.006) would render none of the three nominally significant range-of-motion differences (abduction, internal rotation, external rotation) statistically significant; this is noted explicitly where these results are reported.

## 3. Results

Of 110 patients screened, 25 fulfilled all eligibility criteria and were included in the final analysis (Figure 1). The cohort comprised 11 women (44%) and 14 men (56%), with a mean age of 50.9 ± 13.2 years (range 23–72 years). Mean follow-up was 85.4 ± 34.1 months (range 36–156 months). The dominant extremity was involved in 13 patients (52%). The fracture was classified as Neer three-part in 14 patients (56%) and four-part in 11 patients (44%).

Osteonecrosis of the humeral head was identified by radiography and MRI in 12 of 25 patients (48%), comprising 6 of 14 three-part fractures (43%) and 6 of 11 four-part fractures (55%); this difference was not statistically significant (*p* = 0.69). Comparisons between patients with and without osteonecrosis are summarised in Table 1. Shoulder abduction, internal rotation, and external rotation were each lower in the osteonecrosis group. Mean abduction was 96.7 ± 27.1° versus 121.8 ± 29.2° (mean difference −25.1°, 95% CI −47.9 to −2.3; *p* = 0.031). Mean internal rotation at 90° abduction was 48.3 ± 15.9° versus 66.4 ± 15.5° (mean difference −18.1°, 95% CI −32.1 to −4.1; *p* = 0.014). Mean external rotation was 36.7 ± 16.7° versus 51.8 ± 26.4° (mean difference −15.1°, 95% CI −29.5 to −0.7; *p* = 0.041). None of these three comparisons remained below the Bonferroni-corrected threshold for eight comparisons (*p* < 0.006), and they are accordingly interpreted as a consistent pattern across motion planes rather than as independently confirmed findings (Section 2.5). Constant score, ASES score, and VAS pain score did not differ significantly between groups (Table 1).

Among the twelve patients with osteonecrosis, five had total necrotic angles <100°, four had angles between 100° and 200°, and three had angles >200° (Table 2). The distribution of Cruess classification stages among the 12 patients with osteonecrosis is presented in Table 3. Patients with larger lesions demonstrated significantly higher Cruess stages (*p* = 0.012). No statistically significant differences in shoulder range of motion, Constant score, ASES score, or VAS pain score were observed across subgroups; however, given subgroup sizes of five, four, and three patients, these analyses are descriptive and should not be interpreted as evidence of equivalence (Table 2).

**Figure 3:**
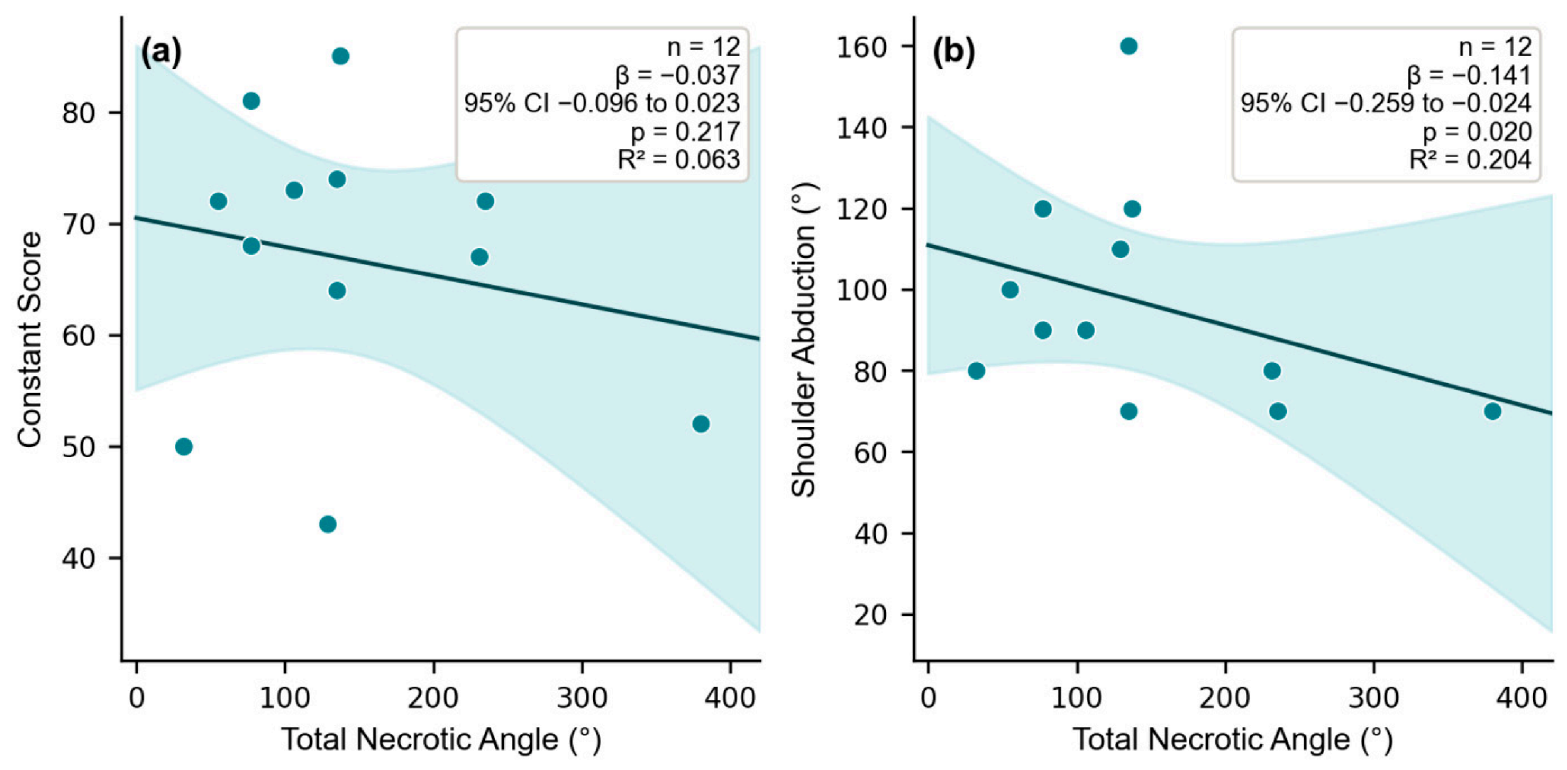
Scatter diagrams illustrating the relationship between total necrotic angle (degrees) and clinical outcomes among patients with confirmed osteonecrosis (*n* = 12). (**a**) Total necrotic angle versus Constant score (β = −0.037; 95% CI −0.096 to 0.023; *p* = 0.217; R^2^ = 0.063). (**b**) Total necrotic angle versus shoulder abduction (β = −0.141; 95% CI −0.259 to −0.024; *p* = 0.020; R^2^ = 0.204). Filled circles represent individual patients; the solid line shows the linear regression fit; and the shaded region represents the 95% confidence interval band.

Linear regression analyses were performed among patients with confirmed osteonecrosis (*n* = 12). Results are presented in Table 4. A greater total necrotic angle was associated with reduced shoulder abduction (β = −0.141; 95% CI −0.259 to −0.024; *p* = 0.020; R^2^ = 0.204) (Figure 3). A clinically meaningful interpretation is that each 50° increase in total necrotic angle was associated with a predicted reduction of approximately 7.1° in shoulder abduction. A negative trend was also apparent for Constant score (β = −0.037; *p* = 0.217; R^2^ = 0.063); however, this did not reach statistical significance. In a sensitivity model incorporating follow-up duration as an additional covariate, the association between total necrotic angle and shoulder abduction remained significant (β = −0.138; 95% CI −0.261 to −0.015; *p* = 0.031).

## 4. Discussion

This study examined MRI-defined humeral head osteonecrosis extent after locked plate fixation of complex proximal humeral fractures and its association with radiographic severity and clinical outcomes at long-term follow-up. Three principal observations emerged. First, osteonecrosis was present in nearly half the patients. Second, its presence was associated with reduced shoulder motion, particularly abduction and rotation, whereas composite functional outcome scores were less markedly affected. Third, among patients with osteonecrosis, greater MRI-defined necrotic lesion extent was associated with higher radiographic Cruess stages and lower shoulder abduction.

The 48% osteonecrosis prevalence observed here lies at the upper boundary of rates published for locked plate fixation of proximal humeral fractures [5,6,7,8,11]. This likely reflects the deliberate restriction of the cohort to Neer three- and four-part fracture injury patterns recognised for their elevated risk of vascular compromise [5]. The extended mean follow-up exceeding seven years may also have facilitated detection of osteonecrotic changes that remain radiographically occult during earlier evaluations.

Shoulder motion was reduced in patients with osteonecrosis compared with those without, a pattern consistent with prior series [9,10]. Yet Constant and ASES composite scores did not differ significantly between groups. This dissociation suggests that patients may compensate for restricted range of motion through preserved strength, adaptation to chronic discomfort, or activity-modification mechanisms that can partially sustain composite scores even as structural integrity deteriorates. The results should be interpreted as associations rather than causal relationships; the cross-sectional design at a single time point cannot establish that osteonecrosis caused the motion restriction.

A distinguishing feature of this study is the quantitative MRI-based measurement of lesion extent. The combined necrotic angle approach, adapted from the Kerboul method originally validated for femoral head osteonecrosis [12], was applied to the humeral head by Sakai et al. [13], who demonstrated that larger MRI-defined necrotic angles were predictive of humeral head collapse. The present study extends this observation to the functional domain: larger necrotic angles were associated with reduced shoulder abduction, with each 50° increase corresponding to an estimated 7.1° reduction in abduction. This magnitude, though modest, may represent clinically meaningful motion loss when cumulative across large lesions. Importantly, this association was independent of follow-up duration in a sensitivity model, providing some support for its robustness. Sakai et al. employed a similar angle-based method but focused on collapse prediction rather than functional outcomes; the present findings add a functional dimension to that structural measure.

The absence of a significant association between necrotic angle and Constant score is noteworthy. The Constant score integrates pain, activities of daily living, range of motion, and strength into a composite, which may dilute motion-specific signals. The finding that abduction but not the composite score associates with necrotic extent may reflect genuine motion-specific impairment rather than global functional decline, or alternatively, may indicate that the composite score is insufficiently sensitive to detect differences in this population.

From a clinical standpoint, quantitative MRI characterisation may provide structural information that complements Cruess staging. A larger necrotic angle, independently of stage, may identify patients at greater risk of progressive motion loss. Whether this information should modify surveillance frequency, activity restrictions, or the threshold for conversion to arthroplasty cannot be determined from this study. Future prospective work with serial MRI and defined decision points will be needed to translate these findings into clinical algorithms.

### Limitations

This study has several important limitations. The sample was small (*n* = 25; *n* = 12 with osteonecrosis), which substantially limits statistical power. The three subgroup ANOVA analyses in Table 2 (*n* = 5, 4, 3) are severely underpowered and should be regarded as descriptive and hypothesis-generating only. The regression analyses were performed on *n* = 12 and provide modest R^2^ values (0.063–0.204); a fully adjusted multivariable model with *n* = 12 would risk overfitting, and the reported simple regression findings indicate that total necrotic angle explains only a portion of variance in outcomes; unmeasured confounders including fracture severity, reduction quality, implant positioning, bone mineral density, and soft-tissue factors likely account for substantial residual variance.

The study is best characterised as ambispective and cross-sectional: records were reviewed retrospectively, but patients were prospectively recalled for a single assessment. This design cannot establish temporal precedence or causal direction between osteonecrosis extent and motion outcomes. The wide range of follow-up (36–156 months) means that patients were assessed at different stages of disease evolution.

Selection bias is a significant concern. Of 110 patients identified, 63 (57%) declined participation or could not be contacted. Patients willing and able to return may differ systematically from non-participants, potentially biasing the cohort towards those with better outcomes or more motivated individuals. This limits generalisability.

The case collection window closed in December 2022 because the study was designed to prospectively enrol patients following IRB approval (granted 27 May 2022), with data collection completed within that calendar year. Extending enrolment beyond December 2022 would have required a protocol amendment and renewal of IRB approval, which was outside the scope of the current study. This is acknowledged as a limitation for future iterations.

Inter-observer ICC for necrotic angle measurements ranged from 0.851 to 0.864 (calculated on the 12 patients with confirmed osteonecrosis), indicating good-to-excellent agreement; however, residual artefact from the locking plate remains a source of measurement uncertainty, and the MARS protocol was not available for all patients.

## 5. Conclusions

Post-traumatic humeral head osteonecrosis after locked plate fixation of Neer three- and four-part proximal humeral fractures was identified in nearly half of patients at long-term follow-up and was associated with significantly reduced shoulder motion, most prominently in abduction and rotational arcs, while global composite functional scores were less clearly affected. Larger MRI-defined necrotic lesions correlated with more advanced radiographic Cruess staging and lower shoulder abduction values. These findings suggest that quantitative MRI assessment of osteonecrosis extent provides clinically relevant structural information that complements conventional radiographic classification. Prospective studies in larger cohorts employing standardised MRI protocols are warranted to confirm these associations and to clarify the role of quantitative MRI characterisation in post-operative monitoring and treatment planning for this patient population.

## Figures and Tables

**Table 1:** Comparison of demographic, functional, and range-of-motion variables between patients with and without osteonecrosis.

Variable	Osteonecrosis (+) *n* = 12	Osteonecrosis (−) *n* = 13	Mean Difference (95% CI)	*p*-Value
Age (years)	48.9 ± 12.8	52.7 ± 13.6	−3.8 (−14.3 to 6.7)	0.462
Follow-up (months)	88.3 ± 36.2	82.7 ± 32.4	5.6 (−22.8 to 34.0)	0.684
Constant score	68.4 ± 13.9	75.6 ± 10.8	−7.2 (−17.9 to 3.5)	0.167
ASES score	72.1 ± 14.7	80.5 ± 12.1	−8.4 (−19.8 to 3.0)	0.138
VAS pain score	2.8 ± 1.7	1.9 ± 1.4	0.9 (−0.4 to 2.2)	0.176
Abduction (°)	96.7 ± 27.1	121.8 ± 29.2	−25.1 (−47.9 to −2.3)	0.031 *
Internal rotation (°)	48.3 ± 15.9	66.4 ± 15.5	−18.1 (−32.1 to −4.1)	0.014 *
External rotation (°)	36.7 ± 16.7	51.8 ± 26.4	−15.1 (−29.5 to −0.7)	0.041 *

Values are mean ± SD. Mean difference = Osteonecrosis (+) minus Osteonecrosis (−). * *p* < 0.05. ASES, American Shoulder and Elbow Surgeons score; VAS, visual analogue scale; SD, standard deviation.

**Table 2:** Comparison of radiographic severity, shoulder range of motion, and functional outcomes across total necrotic angle subgroups (*n* = 12).

Variable	<100° (*n* = 5)	100–200° (*n* = 4)	>200° (*n* = 3)	Mean Difference (95% CI)	*p*-Value
Constant score	74.8 ± 10.4	67.5 ± 13.2	58.7 ± 14.5	16.1 (−13.0 to 45.2)	0.236
ASES score	78.2 ± 11.5	70.1 ± 13.6	62.3 ± 16.8	15.9 (−18.1 to 49.9)	0.281
VAS pain score	2.0 ± 1.2	2.9 ± 1.5	3.7 ± 2.1	−1.7 (−6.1 to 2.7)	0.314
Abduction (°)	112.0 ± 24.1	96.3 ± 18.4	73.3 ± 11.5	38.7 (7.6 to 69.8)	0.071
Internal rotation (°)	58.0 ± 13.5	46.3 ± 11.1	36.7 ± 15.3	21.3 (−8.8 to 51.4)	0.108
External rotation (°)	45.0 ± 17.1	35.0 ± 12.9	23.3 ± 10.4	21.7 (−2.2 to 45.6)	0.174
Cruess stage	2.0 ± 0.7	3.0 ± 0.8	4.3 ± 0.6	—	0.012 *

Values are mean ± SD unless otherwise stated. Mean differences with 95% CI are reported for continuous variables between the <100° and >200° subgroups (descriptive only; *n* = 5 and *n* = 3, respectively). * *p* < 0.05 (one-way ANOVA). Subgroup analyses are reported descriptively; groups of *n* = 5, *n* = 4, and *n* = 3 are severely underpowered, and results should not be interpreted as evidence of equivalence.

**Table 3:** Distribution of Cruess classification stage among patients with osteonecrosis (*n* = 12).

Cruess Stage	Number of Patients	Percentage (%)
Stage I	2	16.7
Stage II	3	25.0
Stage III	4	33.3
Stage IV	2	16.7
Stage V	1	8.3

Values are number of patients (percentage) among the 12 patients with confirmed osteonecrosis.

**Table 4:** Linear regression models for the association between total necrotic angle and clinical outcomes among patients with osteonecrosis (*n* = 12).

Dependent Variable	β	95% CI	*p*-Value	R^2^
Constant score	−0.037	−0.096 to 0.023	0.217	0.063
Abduction (°)	−0.141	−0.259 to −0.024	0.020 *	0.204
Abduction (°), follow-up adjusted	−0.138	−0.261 to −0.015	0.031 *	0.218

All models fitted among patients with osteonecrosis (*n* = 12). β, unstandardised regression coefficient; CI, confidence interval; R^2^, coefficient of determination. * *p* < 0.05.

## Data Availability

The datasets generated and/or analysed during the current study are available from the corresponding author on reasonable request.

## List of Abbreviations

AVN, avascular necrosis; ASES, American Shoulder and Elbow Surgeons score; VAS, visual analogue scale; MRI, magnetic resonance imaging; ORIF, open reduction and internal fixation; CI, confidence interval; ICC, intraclass correlation coefficient.
